# Frequency and Local Etiological Factors of Impaction of Permanent Teeth among 1400 Patients in a Greek Population

**DOI:** 10.3390/dj10080150

**Published:** 2022-08-11

**Authors:** Kalliopi Siotou, Maria-Panagiota Kouskouki, Isidora Christopoulou, Apostolos I. Tsolakis, Ioannis A. Tsolakis

**Affiliations:** 1 Department of Orthodontics, School of Dentistry, National and Kapodistrian University of Athens, 11527 Athens, Greece; 2 Department of Metabolic Bone Diseases, Medical School, National and Kapodistrian University of Athens, 11527 Athens, Greece; 3 Department of Orthodontics, Case Western Reserve University, Cleveland, OH 44106, USA; 4 Department of Orthodontics, School of Dentistry, Aristotle University of Thessaloniki, 54124 Thessaloniki, Greece

**Keywords:** impacted permanent teeth, frequency of impaction, etiology of impaction, eruption disturbances

## Abstract

Background: The purpose of this study is to analyze the frequency of impaction of permanent teeth, beyond the third molars, and to highlight the factors causing this condition. Methods: Panoramic radiographs of 1400 patients that sought orthodontic treatment in private practice were retrieved and examined. All teeth that had not been erupted at the time of the examination while their root formation was completed were considered impacted. Results: In total, 212 out of 1400 patients had at least one impacted tooth (15.14%). The highest incidence of tooth impaction was in the canines of the maxilla, followed by the central incisors of the maxilla, the second molars of the mandible and the second premolars of the mandible. The most common etiological factors responsible for the impaction were the ectopic eruption pathway, loss of space in the arch, the ankylosis of the deciduous teeth and the presence of supernumerary teeth. Conclusions: Tooth impaction is frequently seen in everyday orthodontic practice. The upper canines are the teeth most frequently associated with impaction and failure of eruption. It is important to diagnose cases of impaction early on and identify the etiological factors in order to achieve immediate and effective treatment per patient.

## 1. Introduction

Eruption is a procedure characterized by the axial movement of the tooth from its developmental position in the alveolar bone to its functional site in the occlusal plane [1,2,3]. The term failure of eruption is used to describe the cessation of the eruption of a tooth in the jaw after a period of active eruption [4]. Normal eruption of teeth can be disturbed due to various factors such as mechanical obstacles, the displacement of the dental sperm, odontomas, ankylosis and medical syndromes such as cleidocranial dysostosis and hypothyroidism [5]. One or multiple teeth of primary or permanent dentition can be affected and depending on its etiology, it can lead to complete or partial failure of tooth eruption [6].

The complete failure of eruption leads to impaction, and it can be associated with the existence of a physical barrier or the irregular site of the tooth [7,8]. When the eruption process is not disturbed by a detectable physical obstacle and the tooth is placed in a normal position before emergence, it is primarily retained [8]. In contrast, a tooth that fails to erupt after it has entered the oral mucosa, without a physical obstacle and not due to an abnormal position, is secondarily retained [6,8].

When reviewing the published literature, retention and impaction are frequently used as synonyms, but these disorders differ in their etiology. The causative factors responsible for impaction can be generalized or local. Generalized factors are associated with systematic disorders and syndromes [8] including cleidocranial dysostosis, Down’s syndrome, amelogenesis imperfecta, osteopetrosis and achondroplasia [9]. Among the local factors involved in the failure of eruption are the following: lack of space, supernumerary teeth, developmental point of the root [2], odontogenic cysts and tumors including odontoma, ankylosis, existence of alveolar cleft and idiopathic factors such as primary failure of eruption (PFE) [9]. Local factors producing mechanical obstruction are most frequently associated with the failure of eruption of permanent teeth [10]. Concerning primary retention, a disruption in the dental follicle is the most possible etiologic factor, while the main reason leading to secondary retention is thought to be ankylosis [2].

In the literature, the most common causes that lead to complete failure of eruption are: odontomas, supernumerary teeth, lack of space, ankylosis and primary failure of eruption. Odontoma is the most frequent odontogenic tumor of the oral cavity and the second most frequent tumor of the jaw which can create an obstacle in the eruption of teeth [11].

Even though the causative factors of odontoma are vague, local trauma, genetic and inflammatory factors have been proposed [11]. Odontoma is composed of dental tissue and usually evolves slowly without aggressive development [12]. The two main categories of odontomas are the complex and the compound type. Radiographically, the appearance of odontomas has a great variety [13]. The anterior maxilla is the most common site for compound odontoma, while the complex type seems to mostly affect the posterior mandible. However, odontomas have also been found in soft tissues [11]. Supernumerary teeth are also associated with failure of eruption. The mesiodens is the most frequent supernumerary tooth [14,15,16] and typically is defined as supernumerary tooth in association with the maxillary central incisors [14]. The main effects of the presence of a mesiodens are impaction (frequency 26–52%) and ectopic eruption (frequency 28–82%) of the permanent maxillary central incisors. Other feasible consequences are the lack of the perimeter of the arch, cyst development surrounding the impacted tooth and resorption of the roots of the neighboring teeth. There is also an association between impaction or delayed central incisor eruption and following the impaction of maxillary canine. The treatment of impacted teeth contains surgical intervention, usually combined with orthodontic treatment [14]. Another common etiologic factor of impaction is the lack of space in the arch [2] due to dental crowding or maintenance of the deciduous teeth. Histologically, ankylosis is defined as the fusion of cementum to bone in at least one area, leading to the loss of the periodontal ligament space. Fusion of tooth root and the bone results in a vertical stagnation of eruption, leading to infra-occlusion or impaction [5,17]. An ankylosed tooth is clinically characterized by a lack of normal mobility and a solid sound during percussion. Radiographically, the periodontal ligament space is absent. However, the misinterpretation of the radiograph may make it difficult to distinguish ankylosis from PFE [3]. The diagnosis of ankylosis can occur at any stage of the eruption process and is commonly based on clinical evidence [10]. Primary failure of eruption is a rare disorder characterized by incomplete tooth eruption, although a clear eruption pathway is present. It causes an initial non-ankylosed tooth to partial or complete failure of eruption, because of a disordered eruption mechanism, probably due to anomalies occurring in the PDL [18]. The disorder mostly affects the posterior teeth, and usually, all teeth located distal to the most mesial affected tooth are also involved. The trademark of PFE seems to be infra-occlusion in the posterior area. This situation is observed principally when PFE affects both sides [18]. According to studies, there is an association between PFE and mutation of the PTH1R gene. This mutation has been observed in multiple family members that are affected by PFE [17].

During childhood, the risk of severe orofacial injuries is high. Studies presented oral trauma as the second most commonly injured area in children below 6 years old. The developing permanent tooth is in the most substantial danger when it is included in the area of trauma. The possible consequences involve the dilaceration of the crown or the root, the displacement of the germ and even impaction and eruption disturbances. The greatness of the impact, the stage of the development, the type of injury and the child’s age when the trauma occurs are significant factors that determine if the permanent tooth becomes impacted. While impaction is considered a severe disturbance after an oral trauma, hypoplasia and crown discoloration of the permanent successor should be considered as mild disturbances [19].

In most cases, clinical and radiographic examinations constitute the diagnostic procedure for impacted teeth. Panoramic, periapical and occlusal are the most widely used diagnostic radiographs [20]. Due to their location and their propensity to follow an irregular eruption path, impacted teeth may be responsible for root resorption and carious lesions of the adjacent teeth [8]. In regard to the treatment of impacted teeth, studies report that the possible approaches include observation, intervention, repositioning and surgical extraction [21]. In the case of an impaction attributed to a physical barrier, the early removal of obstacle usually leads to the spontaneous eruption of the impacted tooth [2,8]. The appropriate treatment of impacted teeth should be planed after clinical examination, depending on the position of the teeth and after the investigation of the possible negative effects on the adjacent teeth [20].The aim of the present study is to evaluate the frequency of impaction in 1400 orthodontic patients to assess the most frequently impacted permanent teeth and to investigate the local etiological factors of impaction.

## 2. Materials and Methods

For this study, panoramic radiographs of 1400 patients that sought orthodontic treatment in a private practice were retrieved. The study sample consisted of both children and adolescents. No restrictions concerning the age or the ethnicity of the participants were placed. The exclusion criteria were the systemic disorders, syndromes and craniofacial anomalies such as cleft lip or/and palate as well as previous orthodontic treatment.

A tooth was considered impacted when its eruption was disturbed by a physical obstacle or when its eruption path was interrupted, while the development of the root could not explain the delay of eruption. The examination of the radiographs aimed to the determination of the local factors contributing to impaction. For the causative factors, we considered the following: ankylosis, primary failure of eruption, supernumerary teeth, odontogenic tumors such as odontoma and osteoma, ectopic eruption pathway of the impacted tooth, a lack of space in the arch, trauma or unknown etiology. The only measure of impaction diagnosis was panoramic radiograph. Studying the frequency of impacted third molars was excluded from our study. 

In order to properly determine the exact etiological factor of impaction each radiographic examination was matched with the patient’s intraoral photos and clinical examination, all retrieved by the private orthodontic office. The full orthodontic pre-treatment record of each patient was examined along with the panoramic radiograph. Blinding of the participant names was performed. Three assessors (KS, MK, IC) assessed the records independently. Every disagreement was solved by discussion or consultation with the other two authors (AIT, IAT).

## 3. Results

There were no differences between the results of the three examiners when assessing the radiographs. From the total 1400 patients included in the study, 713 were males (50.93%) and 687 were females (49.07%). The age range of the males was 17–37 and the mean age was 23, while for the females the age range was 16–40 and the mean age was 22. From the analysis of the radiographs combined with the clinical examination and the intraoral photos of the patients, at least one impacted tooth was diagnosed in 212 patients (15.14), while the total number of impacted teeth was 316. Table 1 illustrates the findings concerning impaction in the maxilla and the mandible, and the percentage of each tooth type in relation to the overall number of impacted teeth. The frequency of local causative factors responsible for impaction is demonstrated in Table 2. Table 2 also shows the most frequently affected tooth for each etiological factor. Based on our sample, the most frequently impacted teeth were the maxillary canines (32.28%), followed by maxillary central incisors (17.41%), mandibular second molars (11.39%) and mandibular second premolars (11.08%). In total, 7.91% of impacted teeth were maxillary second premolars, followed by maxillary lateral incisors (4.43%), maxillary second molars (4.11%) and maxillary first premolars (3.48%). The lowest frequency of impaction was presented in mandibular lateral incisors, with 1 incidence out of 316 cases (0.32%). Maxillary impacted teeth consisted of the 71.52% of the total impacted teeth with 226 out of 316 impactions, while impactions of the mandible were the 28.48% (90 impactions).

In the maxilla, the canines had the highest impaction, with 102 of 226 impactions (45.13%). The second most commonly impacted teeth were the central incisors (24.34%), followed by second premolars (11.06%) and lateral incisors (6.19%). Among the impacted teeth of the mandible, second molars presented the highest incidence (40%), followed by second premolars (38.89%), canines (7.78%) and first premolars (5.56%).

**Figure 1:**
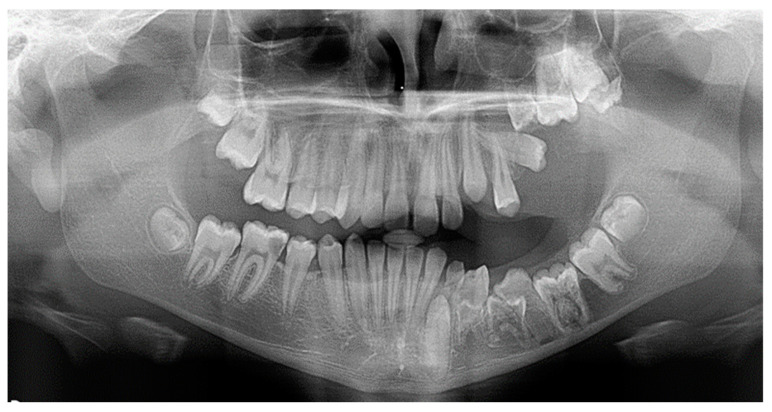
Primary failure of eruption of upper and lower molars.

**Figure 2:**
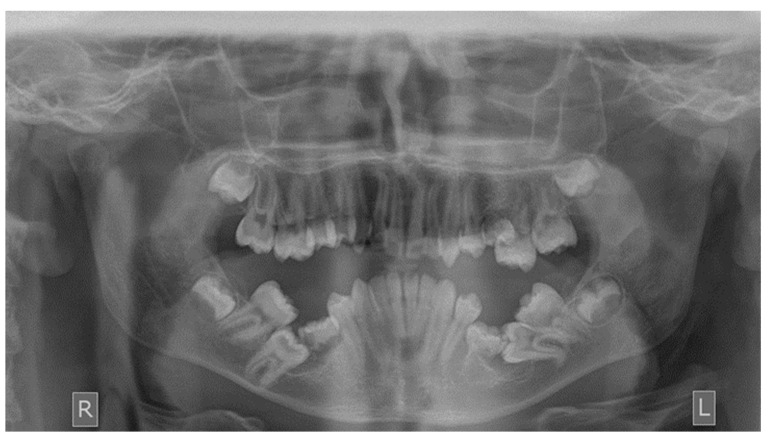
Impacted first mandibular molars.

**Figure 3:**
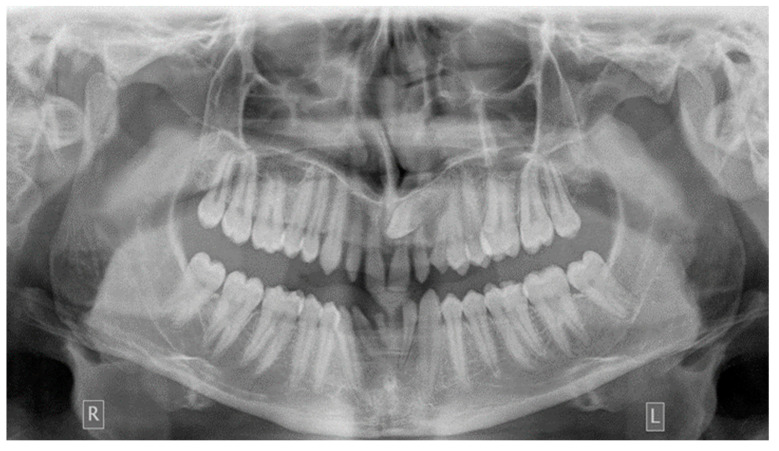
Ectopic eruption of upper left canine.

**Figure 4:**
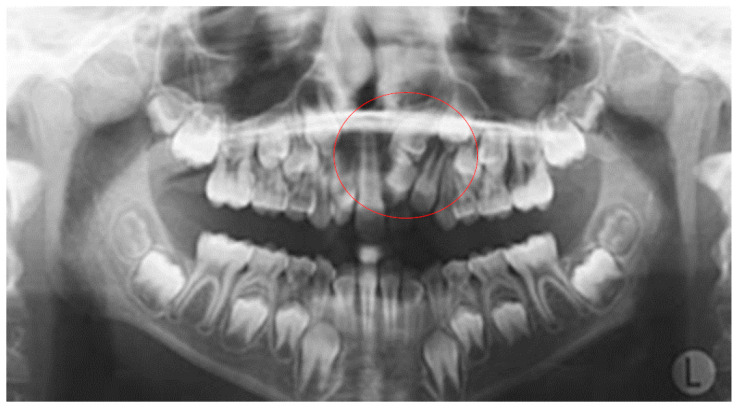
Odontoma in the anterior maxilla.

As far as the etiology of impaction is concerned, 205 patients presented one factor responsible for impaction, while 7 presented two. Table 2 shows the ectopic eruption path as the most frequent factor that was reported in 5.29% of total patients examined and mostly reported in maxillary canines. The next most common etiology was the loss of space in the arch (2.50%), followed by the ankylosis of deciduous teeth (1.71%). Concerning the rest of the causative factors, supernumerary teeth other than deciduous were observed in 1.29% of the patients, while the mesiodens was the most frequent supernumerary permanent tooth. In total, 1.21% of the sample presented tumors such as odontoma and osteoma, more often in association with maxillary central incisors. Supernumerary deciduous teeth affected 0.86% of the patients examined, while the ankylosis of permanent teeth was presented in 0.79 of the cases. The maxillary second premolars were the most frequently impacted teeth due to ankylosis. The least common etiology of impaction proved to be the primary failure of eruption, with 3 reported cases out of 212 patients, that following the radiographic examination were all confirmed by genetic tests. Panoramic radiographs indicated for some local etiological factors are presented below (Figure 1, Figure 2, Figure 3 and Figure 4).

More specifically, the ankylosis and the loss of space, which cannot be detected only via panoramic radiograph were also assessed by the intraoral photos and the clinical examination provided by the private orthodontic office. The following signs of ankylosis were considered: infra-occlusion, dull percussion sound, mesial drift of adjacent teeth and mobility tests. The examination concerning the percussion sound was performed by tapping the tooth vertically as well as horizontally with the handle of a probe. High and dull sounds were recorded and the adjacent teeth served as controls. The loss of space was firstly examined radiographically and afterwards confirmed and measured clinically. Trauma as a possible etiological factor was assessed radiographically and by the dental record of the patient (history of previous trauma as reported by the parents and/or the pediatric dentist/dentist and radiographic examination at the time of the incident).

## 4. Discussion

Tooth impaction is a common issue which orthodontists will have to be able to diagnose early on and treat with caution [1]. In this study, we investigated the frequency of impaction in a sample of 1400 orthodontic patients of a private office in Greece. The patients were children and adolescents and our study focused on estimating the frequency of impacted permanent teeth and the most frequent local etiological factors related with impaction.

Deviations in the frequency of impaction in various studies may be attributed to differences in the sample, diagnostic procedure and methodology used. In reviewing the literature, the frequency of tooth impaction in the general population varies between 2.9% and 18.8% [1,21]. Drenski et al. [22] reported 6.3% incidence of impaction in a sample of 506 panoramic radiographs of Croatian adolescent orthodontic patients. This frequency is lower compared to ours (15.14%) and may be due to difference in the sample size and the age of the patients, since we included both children and adolescents. Uslu et al. [23] and Gupta et al. [24] also reported a lower incidence of impaction (2.9% and 3.74%, respectively) in their samples of 900 Turkish and 1123 Indian patients, respectively.

In our study, the most commonly impacted tooth was the maxillary canine, with a frequency of 32.28% of total impactions. This finding is in accordance with the findings of similar radiographic studies published in the literature. Wolf and colleagues [20], Gupta and colleagues [24], Frank [21], Sajnani [9] and Otsuka and colleagues [25] also reported that maxillary canines are the most frequently impacted permanent teeth apart from third molars. This can be relative to the fact that they erupt last in the upper arch and due to lack of space, they often remain impacted.

The main difference in our study compared to previous ones concerns the impaction of mandibular second molars (2.57% of total sample), which were the third most frequently impacted teeth after maxillary canines and maxillary central incisors. According to Rubin and colleagues [26], the frequency is 0.05% in the general population and 1% in the orthodontic population. Palma and colleagues [2] also describe the failure of eruption of second permanent molars as rare, with an incidence of 0.06% in the normal population. Our results are also higher than those of Cassetta and colleagues [27], who found 57 impacted mandibular second molars in a sample of 2945 Caucasian young orthodontic patients (1.94%). This difference could be attributed to the different size of the samples and the age of the participants.

The frequency of impacted mandibular canines was found to be 0.5% among 1400 patients in the current study, which is higher than the study by Jain and colleagues [28], where the frequency was estimated as 0.37% in a sample of 1593 Indian patients. Aydin and colleagues [29] reported a frequency of 0.44% among 4500 patients of the Turkish population. In our study, mandibular canines represented the 2.2% of 316 impactions, which is lower compared to the study of Al-Abdallah and colleagues [1], where the frequency was 8.1% among 297 impacted teeth. 

In the present study, 226 out of 316 impacted teeth were in the maxilla (71.52%). This percentage is higher compared to the study of Al-Abdallah et al., where the impacted teeth of the maxilla were 185 out of 297 total impactions (62.29%) [1]. The age of the included patients was between 15 and 40 years. In contrast, the reported frequency by Drenski et al. was 78.57% for maxillary impacted teeth in a sample of adolescents with 42 impactions in total [22]. 

Concerning the etiology of impaction, ectopic eruption path was our study’s most frequent local etiological factor. Maxillary canine was the most affected tooth, justified also by the results of previous studies [23,24]. The second most common etiologic factor was the loss of space in the arch, followed by the ankylosis of deciduous teeth and supernumerary permanent teeth. The least common cause of impaction proved to be the primary failure of eruption, which was present in the 0.21% of the total sample of panoramic radiographs.

Loss of space in the arch was responsible for impaction in 2.5% of the present sample, while maxillary canines were the most commonly impacted teeth. However, Palma et al., in their study of 26 patients between 7 and 17 years of age, reported permanent upper and lower second molars as the impacted teeth most influenced by the lack of space [2]. However, there is a significant difference in the size of the two samples, which may explain the different results.

Regarding odontoma, it has been stated that the anterior maxilla is the most common site for its development [30], in accordance with the results of our study, where maxillary central incisors were most frequently involved. Isola and colleagues (2017) [11], during their study among 45 patients with odontomas, reported maxillary central incisors and canines as the most affected teeth.

Among supernumerary teeth, mesiodens has the highest frequency in the general population [14,15,16]. Single or multiple mesiodens may appear. Spontaneous eruption of the impacted central incisor is thought to be likely after the early removal of a mesiodens (if the space needed is available). The possibility of eruption is also influenced by the eruptive capacity of the central incisor, which is strongly associated with the root development and the age of the patient. Early diagnosis and post-surgical follow-up are considered to be indispensable when a mesiodens is present [14]. Mesiodens was also the most common supernumerary tooth in our study. 

According to Bhuvaneswarri et al. (2018), although ankylosis can be detected in both permanent and primary dentition, deciduous molars are the most affected teeth [10]. In the present study, the ankylosis of deciduous teeth was found in 0.79% of our patients, while the ankylosis of permanent teeth was reported in 1.71%. Mandibular second premolars were the most commonly impacted-ankylosed teeth.

Primary failure of eruption is a rare disorder with a frequency between 0.01% and 0.06% in the general population [18,31]. There is usually difficulty in differential diagnosis between PFE and other eruption disorders, such as ankylosis [18]. The difficulty exists primarily because both PFE and ankylosis have clinical and developmental similarities [17]. In our study, 0.21% of the patients were diagnosed with primary failure of eruption, with the disorder affecting the posterior permanent teeth, in agreement with previous studies focusing on the same anomaly [6,18,31].

The frequency of oral trauma in children under 6 years old is considered to be 17%, based on the epidemiological studies. Depending on the circumstances of the injury and the patient’s age, the trauma may result in the impaction of the permanent tooth [19]. According to the current study, trauma was responsible for impaction in eight patients, which is 0.57% of the total children and adolescents examined. The highest incidence of impaction was observed in maxillary incisors. Early diagnosis combined with long-term examination are crucial in case the clinician expects severe disturbances after the trauma. Most disorders will be revealed after the eruption of the permanent incisors [19].

**Strengths**

The strengths of the present retrospective study include using a methodology following well-established guidelines. The panoramic radiographs were all analyzed independently by three assessors (KS, MK, IC). Any disagreements regarding the findings were solved by discussion or consultation with the fourth and fifth author (AIT, IAT). The radiographic findings were all confirmed and associated with the full orthodontic pre-treatment record of each patient, retrieved by the private orthodontic office. More specifically, following a meticulous analysis, each radiographic examination was matched with the patient’s intraoral photos and clinical examination. At this point it should be highlighted that several similar studies have been published in the literature. However, this is the first study with such a large patient sample. Moreover, it is the first time that so many and different probable etiological factors for impaction are examined in the same study. To be more specific, the clinical relevance of the present study is to estimate the frequency and the local etiological factors of impaction and highlight the usefulness of the statistical data to facilitate timely detection of impacted teeth aiming to the properly design and execution of the treatment plan.

**Limitations**

This study aimed to investigate the frequency of tooth impaction due to local factors in children and adolescents who sought orthodontic treatment and demonstrate the frequency of treatment of impacted teeth in the daily orthodontic practice. The limitation of this study is the exclusion of the impaction frequency of the third molars. Moreover, syndromic conditions were excluded. Although we did not have restrictions concerning the ethnicity of the patients, most of them were Caucasians, so the ethnicity of participants may also create some bias in the reported results. Another possible limitation of this study may arise from the fact that we diagnosed impaction primarily on the radiographic examination of the patients, although it was matched with the orthodontic clinical record of each patient, which may lead to the underestimation of the results. Nevertheless, as we determine the definition of impaction and related it to root development, we believe that the margin of error is significantly reduced.

## 5. Conclusions

Our study indicates that tooth impaction is a frequent condition that orthodontists will encounter and will have to be able to properly treat. In a sample of 1400 patients, after radiographic examination, impaction was identified in 212 patients (15.14%). With the exclusion of third molars, the most frequently impacted teeth were the maxillary canines (32.28%), while the most common etiologic factor was considered to be the ectopic eruption path, followed by intra-arch loss of space and ankylosis of deciduous teeth. As impaction seems to be a relatively common phenomenon, orthodontists should be aware of the frequency and the causative factors of impaction to properly valuate any delay in eruption and diagnose early incidences of failure of eruption. The immediate and effective personalized treatment is important for the orthodontic movement and preservation of impacted teeth in the dental arch.

## Figures and Tables

**Table 1:** Distribution of impacted teeth.

Tooth Category	Maxilla				Mandible				Total (N + n)	% of Total Impactions
	N	% of Impacted Teeth in Maxilla	% of Total Impacted Teeth	% of Total Patients (1400)	n	% of Impacted Teeth in Mandible	% of Total Impacted Teeth	% of Total Patients (1400)		
Central incisor	55	24.34	17.41	3.93	2	2.22	0.63	0.14	57	18.04
Lateral incisor	14	6.19	4.43	1.00	1	1.11	0.32	0.07	15	4.75
canine	102	45.13	32.28	7.29	7	7.78	2.22	0.50	109	34.49
First premolar	11	4.87	3.48	0.79	5	5.56	1.58	0.36	16	5.06
Second premolar	25	11.06	7.91	1.79	35	38.89	11.08	2.50	60	18.99
First molar	6	2.65	1.90	0.43	4	4.44	1.27	0.29	10	3.16
Second molar	13	5.75	4.11	0.93	36	40.00	11.39	2.57	49	15.51
**Total**	**226**	**100**	**71.52**	**16.16**	**90**	**100**	**28.49**	**6.43**	**316**	**100**

**Table 2:** Frequency of etiological factors of impaction.

Etiological Factor	Frequency	% of Total Panoramic X-rays (1400)	Most Commonly Affected Teeth
Ankylosis	11	0.79	Maxillary second premolars
Ankylosis of deciduous teeth	24	1.71	Mandibular second premolars
Primary failure of eruption	3	0.21	First and second molars
Supernumerary deciduous	12	0.86	Maxillary canines
Supernumerary other than deciduous	18	1.29	Maxillary central incisors
Odontoma-osteoma	17	1.21	Maxillary central incisors
Other obstacles	6	0.43	Maxillary second premolars
Ectopic eruption path	74	5.29	Maxillary canines
Loss of space	35	2.50	Maxillary canines
Trauma	8	0.57	Maxillary incisors
Unknown	11	0.79	Maxillary canines

## Data Availability

Not applicable.
